# Retrospective analysis of clinical laboratory parameters in Han Wistar rat controls

**DOI:** 10.3389/ftox.2025.1684191

**Published:** 2025-12-03

**Authors:** Rupert Kellner, Alexander Amberg, Frank Bringezu, Dragomir Ivanov Draganov, Annika Kreuchwig, Wolfgang Muster, Guillemette Duchateau-Nguyen, Nils Oberhauser, Paolo Piraino, Markus Schaefer, Nelly Simetska, Thomas Steger-Hartmann, Sylvia E. Escher

**Affiliations:** 1 Fraunhofer Institute for Toxicology and Experimental Medicine ITEM, Chemical Safety and Toxicology, Hannover, Germany; 2 Sanofi, Preclinical Safety, Frankfurt, Germany; 3 Merck Heathcare KGaA, Darmstadt, Germany; 4 Hoffmann-La Roche, Roche Innovation Center, Basel, Switzerland; 5 Bayer Research and Development, Pharmaceuticals, Preclinical Development, Berlin, Germany; 6 Novartis, Basel, Switzerland; 7 Organon SRL, Bucharest, Romania

**Keywords:** Han Wistar rat, preclinical data, clinical chemistry, enzyme activity, hematology, reference intervals, minerals

## Abstract

**Introduction:**

Despite the availability of control animal data sets from toxicological studies, the influence of external factors, such as age of animals, test site and study conditions, on clinical laboratory parameters in rats is only sparsely characterized.

**Objective:**

In order to analyze the covariates of study design, we leveraged the largest available curated collection of control animal data from toxicological studies, sourced from five European pharmaceutical companies. We investigated the influence of external factors on commonly measured clinical chemistry, enzyme activity and hematology parameters in Han Wistar rats of both sexes.

**Materials and Methods:**

457,605 control group clinical laboratory data points from 1,288 legacy toxicity studies on Han Wistar rats were curated and analyzed by ANOVA and partial eta squared to discern their effect sizes.

**Results:**

Our analysis revealed that bodyweight, used as a surrogate for age in rats, significantly influences some parameters, while demonstrating stability in others. Descriptive statistics and tolerance intervals are provided for 20-g body weight class intervals. The effect size of these body weight classes, as calculated by partial eta squared, is large for parameters that change during development (e.g., phosphate or alkaline phosphatase) but was negligible for more stable parameters (e.g., calcium and alanine aminotransferase). For parameters which are less dependent on body weight class, the relative influence of other factors, namely, the company providing the study data, as well as study year is more prominent. These factors likely act as summary factors for various influences such as changes in analytical protocols, diet or housing conditions.

**Conclusion:**

This analysis provides a comprehensive overview of parameter variability and offers critical guidance for parameters which need to be controlled when utilizing historical control data to establish reference intervals or generate virtual control groups.

## 1 Introduction

The collection of existing data from preclinical *in vivo* animal studies, made possible by the collaboration of several companies and its integration into a relational database, represents a significant reservoir of knowledge that can be leveraged to address data and knowledge gaps. In the Innovative Medicine Initiative (IMI) project eTOX (electronic toxicity), aggregated preclinical toxicity data from pharmaceutical companies were shared on a study group basis (Briggs et al., 2012). This was further refined in the follow-up project eTRANSAFE (Enhancing TRANslational SAFEty Assessment through Integrative Knowledge Management), which involved sharing studies at the individual animal level (Sanz et al., 2023). A key subproject within eTRANSAFE focused on harmonizing control group data, intended for randomized selection as virtual control groups (VCGs), ultimately aiming to replace concurrent controls in preclinical toxicity studies (Steger-Hartmann et al., 2020; Gurjanov et al., 2024). For implementing VCGs, understanding the structure of the collected data and their interdependence is crucial. Selection from a broad range of values may lead to increased variance in the virtual controls, hindering the ability to achieve statistically significant results when comparing treatment groups from specific studies that may exhibit a narrower range of variation. Moreover, the accumulated data can provide a more accurate reference for the range of baseline measurements including upper and lower limits of normal.

Clinical parameter measurements in animal studies can vary not only between species and between sexes (Aigner, 2021) but may also be influenced by other factors such as the age of the animals. This variability is particularly notable in young animals, commonly used in short-term rodent studies, where the animals are still in their growth phase. Differences between sexes and between 4- and 13-week toxicity studies have been documented in Sprague-Dawley rats (Petterino and Argentino-Storino, 2006). While individual animal measurements can be traced in legacy studies due to their association with unique identifiers, determining individual ages is much more challenging, as typically only age ranges are available for groups at the start of experiments. Even a small range may make a difference in the short lifespan of young animals. Thus, using individual measurements is preferable for comparative analyses. Given that body weight increases with age, we will use body weight as a surrogate for age, as it is consistently recorded for each animal in a study and holds intrinsic value. Additional factors that may influence clinical parameters include the company and study year, which can serve as proxies for various study-related influences that may be poorly documented or unknown. For instance, the company or site may reflect the effects of specific diets, laboratory personnel, analytical methods, or measurement techniques that cannot be precisely harmonized across different locations. The study year could also indicate genetic drift in laboratory animal stocks if the animals were sourced from the same breeders.

This retrospective study reports on clinical chemistry, enzyme activity and hematology results from control groups in Han Wistar rat toxicity studies, with a primary focus on their relationship to body weight. Both sexes were analyzed separately, which is essential because they differ in their body weight development. For commonly used parameters, descriptive statistics (medians with 10th and 90th percentiles, and [geometric] means with standard deviations) and tolerance intervals for different body weight classes are provided. Their analysis aims to enhance understanding of the parameters that need to be controlled when sampling relevant virtual control groups and provides insights into the variability of clinical chemistry, enzyme activity, and hematology parameters in *in vivo* rat studies. The described tolerance intervals for each parameter can serve as historical controls for comparison with internal data sets.

## 2 Materials and methods

### 2.1 Data collection and curation

Data from toxicological studies were compiled in SEND or SEND-like format by five pharmaceutical companies (Bayer, Merck, Novartis, Roche, and Sanofi) and imported into a common database called the ViCoG (Virtual Control Group) DB. The domains Demographics (DM), Body Weights (BW) and Laboratory Measurements (LB) of ViCoG DB version 1.6 (released on 04 July 2024, to the participating companies) were evaluated. Careful curation and harmonization across the different source companies were essential to ensure comparability and suitability for further analysis. This allowed selection of the same results and measurements in the same unit.

In the BW domain, different spellings in BWTEST (Test Name) were replaced by the standard ‘Body weight’ and ‘Terminal body weight’ and all weights in BWORRES (Result or Findings as Collected) and associated units in BWORRESU (Unit of the Original Result) were converted to grams (g). Body weights served as a surrogate parameter for the age of the animals, as individual ages were rarely reported in the data and could not be determined with sufficient confidence. Available body weights were aligned with laboratory measurements in the LB domain, prioritizing measurements taken on the same day, with consecutive days (±1 day) also accepted.

In the LB Domain, individual measurements are identified by LBTEST (Test or Examination Name), LBCAT (Category for Lab Test) and LBSPEC (Specimen Type), and these entries were harmonized with the aid of LBTESTCD (Test or Examination Short Name) to facilitate the selection of distinct data sets. Differences in description, spelling or grammar (plural vs. singular, bold letters, typos, use of commas, level of provided details, etc.) had to be curated for the effect terms and their specimen. Other curation steps corrected misplacement of, e.g., specimen details in effect terms, while at the same time filling data gaps in the specimen field. Effect terms were taken as far as available from the SEND terminology or were standardized to one most frequently used term. One measure of the success of this curation is the reduction in the number of individual terms per data type. As part of this process, the number of effect terms in the LB domain of the ViCoG DB fell from 821 to 136 terms, while the number of specimen terms fell from 16 to 13 and the number of units from 125 to 24. The 28 evaluated parameters (Clinical chemistry: albumin, bilirubin, calcium, chloride, cholesterol, creatinine, glucose, phosphate, potassium, protein, sodium, triglycerides; Enzyme activity: alanine aminotransferase, alkaline phosphatase, aspartate aminotransferase; Hematology: basophils, eosinophils, erythrocytes, hemoglobin, leukocytes, lymphocytes, MCH, MCHC, MCV, monocytes, neutrophils, platelets, reticulocytes; Table 1) were derived from harmonizing 135 different entries in the original LBTEST collection.

**TABLE 1:** Effect sizes of the three factors body weight (BW) class, company, and study year with controlled influence of the other factors according to partial eta squared; A) clinical chemistry, B) enzyme activity and C) hematology measurements from Han Wistar rats in the ViCoG DB version 1.6 (N number of measurements in the analysis). An abbreviated version of this table on a preliminary dataset (version 1.0 of the ViCoG DB) has been used in a Workshop presentation (Golden et al., 2024).

Parameter		Females	BW class	Company	Year	Males	BW class	Company	Year
		N				N			
A)	Albumin	6095	0.077	0.170	0.205	8739	0.022	0.083	0.214
	Bilirubin	4255	0.021	0.318	0.070	6509	0.013	0.320	0.064
	Calcium	5913	0.004	0.197	0.114	8396	0.006	0.129	0.183
	Chloride	6110	0.002	0.021	0.151	8625	0.026	0.043	0.117
	Cholesterol	5988	0.034	0.039	0.109	8471	0.035	0.026	0.195
	Creatinine	5793	0.045	0.204	0.186	8579	0.081	0.228	0.168
	Glucose	6812	0.017	0.216	0.118	9485	0.020	0.331	0.097
	Phosphate	5677	0.232	0.041	0.069	8228	0.398	0.095	0.040
	Potassium	6541	0.007	0.337	0.137	9315	0.007	0.255	0.114
	Protein	4747	0.161	0.032	0.055	7390	0.154	0.052	0.053
	Sodium	6662	0.003	0.138	0.157	9373	0.013	0.183	0.134
	Triglycerides	5788	0.018	0.303	0.187	8305	0.020	0.198	0.181
B)	Alanine Aminotransferase	5933	0.003	0.157	0.104	8519	0.010	0.124	0.097
	Alkaline Phosphatase	5972	0.307	0.084	0.086	8522	0.396	0.085	0.053
	Aspartate Aminotransferase	5911	0.010	0.005	0.048	8480	0.032	0.012	0.067
C)	Basophils	3809	0.012	0.039	0.126	6921	0.007	0.058	0.087
	Eosinophils	6065	0.002	0.014	0.081	8908	0.040	0.020	0.094
	Erythrocytes	6429	0.028	0.056	0.047	9414	0.229	0.048	0.059
	Hemoglobin	6314	0.014	0.062	0.128	9299	0.077	0.088	0.119
	Leukocytes	6866	0.069	0.016	0.132	10034	0.026	0.040	0.078
	Lymphocytes	6293	0.082	0.014	0.134	9238	0.041	0.036	0.081
	MCH	6253	0.015	0.013	0.207	9241	0.199	0.032	0.163
	MCHC	7646	0.003	0.046	0.267	10815	0.020	0.100	0.228
	MCV	6432	0.031	0.051	0.239	9424	0.310	0.035	0.212
	Monocytes	6124	0.004	0.023	0.181	9055	0.001	0.042	0.142
	Neutrophils	5391	0.002	0.013	0.055	8090	0.009	0.042	0.047
	Platelets	6218	0.049	0.017	0.123	9165	0.074	0.031	0.147
	Reticulocytes	5224	0.068	0.085	0.043	7903	0.229	0.119	0.054

Partial eta squared values in color for large (≥0.14, red) and medium (≥0.06, blue) effects and in gray for values below a small effect (<0.01, gray).

In addition, units needed better standardization and were finally standardized to SI-units. Standardization of measurements in LBORRES (Result or Finding in Original Units) and LBORRESU (Original Units) was achieved through a two-step process. First, units were converted to the nearest prevalent unit by applying factors in the range of 10^-3^ to 10^3^ (e.g., mg/dL to g/L with a factor of 0.01 or 10^10^/L to 10^9^/L with a factor of 10). Special characters and variations in unit spellings were also eliminated in this step. In case of missing values, columns LBSTRESC (Character Result/Finding in Std Format) and LBSTRESU (Standard Units) were utilized. Second, converted units were standardized to SI units using applicable conversion factors (e.g., glucose from g/L to mmol/L with a factor of 5.6). This reduced original 38 nominal units in LBORRESU to seven standardized SI units. About 20% of the original units were already SI units and did not require any further modification.

### 2.2 Data selection

The predominant species in the ViCoG DB is the rat, particularly the Han Wistar strain. Therefore, Han Wistar rats have been selected for this analysis and all commonly taken clinical chemistry, enzyme activity and hematology data were extracted from the curated database. For these tests, there are 780,205 individual measurements in the LB domain of the ViCoG DB, of which 457,605 had associated body weight and study year and were adequately filled for the analysis. The data had been acquired by the five companies within the years 1988–2022 and come from 1,288 legacy toxicity studies. Ethics committees of the companies had reviewed the animal experiments in advance.

Both sexes were analyzed separately due to considerable differences in body weights and potential other sex-specific deviations. Several specimens are reported. Serum measurements were evaluated for the clinical chemistry and enzyme activity parameters, and whole blood was used for hematology. Most data were log-transformed as they clearly showed a right skewed distribution on the original scale. Exceptions are some hematology parameters such as erythrocytes, MCH, and platelets (see results).

Despite careful curation, some outliers or small clusters of measurements far from the rest were identified, which could not be attributed to any reason from the data available in the database. They were usually from single studies where misplaced measurement units, or another unknown factor could have led to the deviation. These values were excluded from the analysis, i.e., bilirubin ≥0.08 mmol/L (gap between 0.06 and 0.09, only males from two companies with values up to 18.8 mmol/L, n = 26), creatinine ≥20 mmol/L (gap between 0.18 and 24, males from one company with values up to 38 mmol/L, n = 7), and protein <10 g/L (one female at 8.6 g/L, gap to 37.4) in clinical chemistry, alkaline phosphatase ≥30 μkat/L (one male at 32.0 μkat/L, gap to 13.7) in enzyme activity, as well as MCH <0.25 fmol (gap between 0.24 and 0.56, only males from three companies with values down to 0.12 fmol, n = 5) and MCHC <5 mmol/L (gap between 4.4 and 15.0, same males as in MCH with values down to 2.3 mmol/L) in hematology.

Cell counts tended to have more outliers, all in the same unit (10^9^/L): basophils <0.001 (gap between 0.0006 and 0.0015, females and males from one study with values down to 0.0001, n = 15) and >5 (one male at 5.7, gap to 0.55), eosinophils <0.005 (gap between 0.0014 and 0.01, females and males from one study with values down to 0.0004, n = 15), leukocytes <0.1 (gap between 0.09 and 0.57, females and males from two companies with values down to 0.026, n = 19) and ≥400 (one male at 443.2, gap to 57.9), lymphocytes <0.08 (gap between 0.07 and 0.1, females and males from one study with values down to 0.02, n = 17), monocytes <0.002 (gap between 0.0012 and 0.01, females and males from one study with values down to 0.0003, n = 17), and neutrophils <0.01 (gap between 0.0096 and 0.11, females and males from one study with values down to 0.004, n = 17). One study contributed all values excluded from the lower border of basophils, eosinophils, lymphocytes, monocytes, and neutrophils, and most of the leukocyte values as well (n = 98).

Overall, 147 values have been excluded, which had been provided by four companies. Apart from the cell counts from one study with more females than males, only one value originated from a female, the others from males.

### 2.3 Subgrouping of data

To analyze the parameters according to the rats’ age development, body weights were classified in 20 g increments. Classes with low occupancy rates were trimmed from both ends to avoid variability in median values due to insufficient animal numbers. This resulted in 8 classes for females (140–300 g) and 16 classes for males (180–500 g). Descriptive statistics for all parameters across these body weight classes are provided in the Supplementary Material, including median values with 10th and 90th percentiles, geometric means with geometric standard deviations for log-transformed data, and means with standard deviations for original scale data, along with upper and lower boundaries of tolerance intervals. The units are as in the respective figures in the results.

### 2.4 Statistical methods

All calculations with the extracted data were performed using R version 4.4 (R Core Team, 2024). Two-sided tolerance intervals were computed with the proportion set to 0.9 and p ≤ 0.05, using the tolerance library (Young, 2010) and log-normal distribution for log-transformed data (geometric means in the supplementary tables) and normal distribution for original scale data (means in the tables). These intervals, with a 95% confidence level, will encompass 90% of the data and can serve as historical controls for comparison with other datasets.

To assess the influence of the factors body weight class, company, and study year on the different measurements as dependent variables, fixed effect model ANOVAs were performed. Residual plots were visually inspected and showed no obvious deviations from homoscedasticity or normality. That approach was further refined using the lme4 library (Bates et al., 2015) to calculate mixed effect model ANOVAs: As fixed effects, body weight class and company (without interaction terms) were entered into the model. As random effects, intercepts for study years and individual animals and no random slopes were used. Likelihood ratio tests of the full model with the effect in question against the model without the effect in question were computed.

To evaluate effect sizes among the three factors, partial eta squared values from the fixed effect model ANOVAs mentioned above were calculated using the lsr library (Navarro, 2015) and type III sum of squares. Partial eta squared estimates the variance explained by a given factor after controlling for the influence of other factors. According to Cohen (1988), effect size thresholds are set at 0.01 for small, 0.06 for medium, and 0.14 for large effects.

## 3 Results

The control group data from 1,288 legacy toxicity studies on Han Wistar rats in the ViCoG DB were collected by five companies from 1988 to 2022.

**FIGURE 1:**
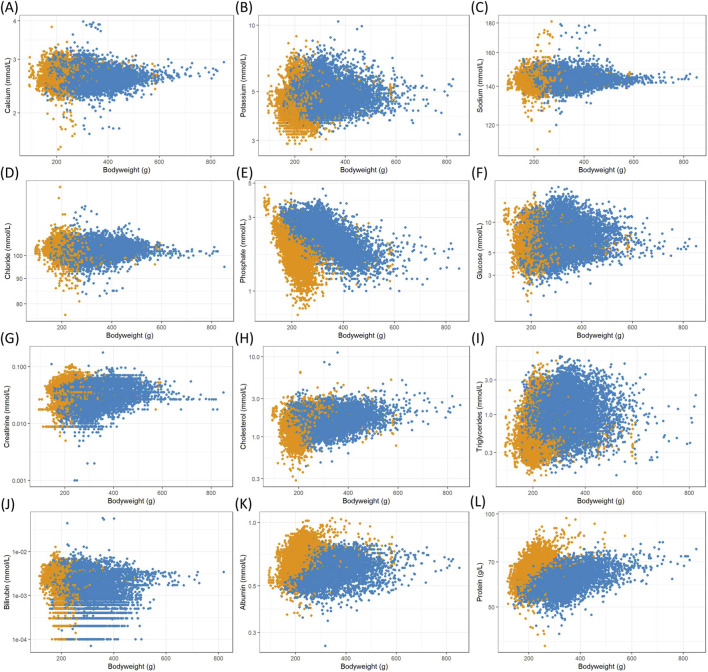
Scatterplots of logarithmized clinical chemistry measurements in serum versus female (orange) and male (blue) body weights from Han Wistar rats in the ViCoG DB; **(A)** Calcium, **(B)** Potassium, **(C)** Sodium, **(D)** Chloride, **(E)** Phosphate, **(F)** Glucose, **(G)** Creatinine, **(H)** Cholesterol, **(I)** Triglycerides, **(J)** Bilirubin, **(K)** Albumin, and **(L)** Protein.

**FIGURE 2:**
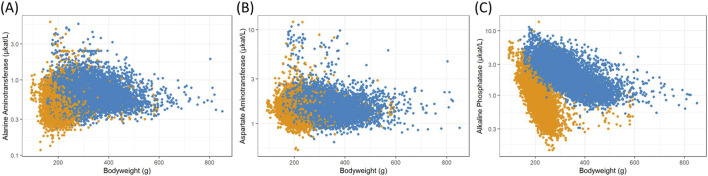
Scatterplots of logarithmized enzyme activity measurements in serum versus female (orange) and male (blue) body weights from Han Wistar rats in the ViCoG DB; **(A)** Alanine aminotransferase, **(B)** Aspartate aminotransferase, and **(C)** Alkaline phosphatase.

**FIGURE 3:**
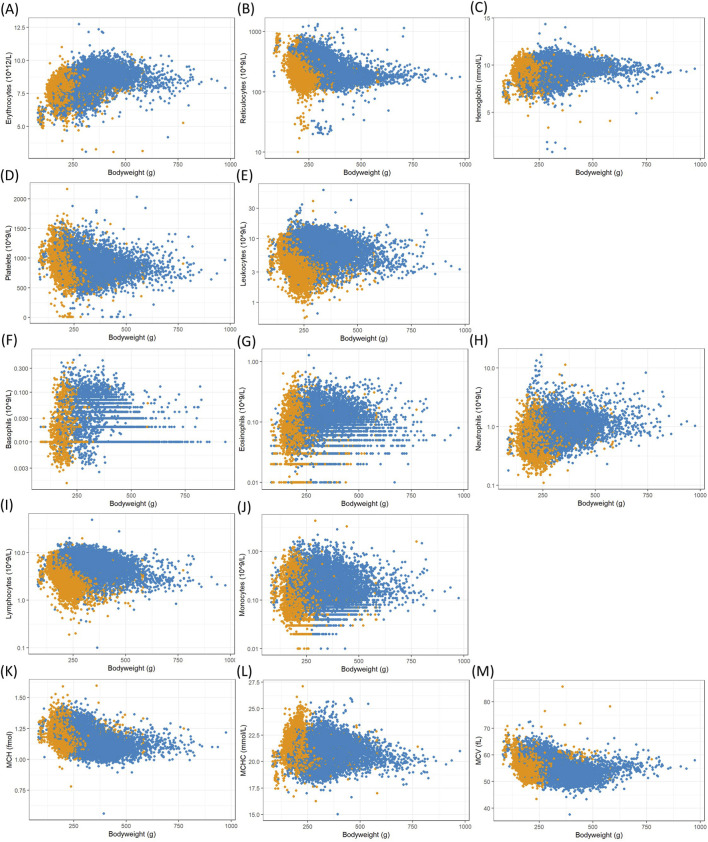
Scatterplots of hematology counts and measurements, mostly logarithmized, in whole blood versus female (orange) and male (blue) body weights from Han Wistar rats in the ViCoG DB; **(A)** Erythrocytes, **(B)** Reticulocytes, **(C)** Hemoglobin, **(D)** Platelets, **(E)** Leukocytes, **(F)** Basophils, **(G)** Eosinophils, **(H)** Neutrophils, **(I)** Lymphocytes, **(J)** Monocytes, **(K)** MCH, **(L)** MCHC, and **(M)** MCV **(A, C, D, K–M)**: original scale).

The resulting data were analyzed with a focus on body weight as a surrogate for the animals’ age, which is typically not recorded individually in these studies. Commonly analyzed clinical chemistry, enzyme activity and hematology measurements from Han Wistar rats collected in the ViCoG database were plotted against individual body weights at the time of measurement to illustrate their growth progress (Figures 1–3). Males typically grow faster and are larger, resulting in a scatterplot shifted towards higher body weights. From that aspect alone, differences between the two sexes are evident. The allocation into 20 g body weight classes resulted in 8 well populated classes for females (140–300 g) and 16 for males (180–500 g). Depending on the different parameters, most classes contained over 100 measurements, with centrally located classes exceeding 1,000 values. To establish robust reference intervals, a minimum requirement of 20 and a recommendation of more than 120 animals have been identified (ASVCP-QALS, 2011). Such high numbers are achievable only by combining data from multiple studies.

The range of values is generally comparable between the sexes although notable deviations occur in some parameters: Higher serum albumin (Figure 1K) and protein (Figure 1L) concentrations are observed in females compared to males, while lower potassium (Figure 1B), phosphate (Figure 1E), and cholesterol (Figure 1H) concentrations as well as alkaline phosphatase (Figure 2C) enzyme activity are observed in females compared to males. Hematology data show less pronounced differences, and lower numbers of leukocytes (Figure 3E), neutrophils (Figure 3H), and lymphocytes (Figure 3I) can be discerned in whole blood of females compared to males.

A few measurements were excluded from the scatterplots (see details in Materials and Methods) as they are considered outliers, likely caused by recording errors. Nevertheless, there are substantial variations from the usual range shown as a dense cloud in the scatterplots especially in the electrolyte measurements calcium (Figure 1A), sodium (Figure 1C), and chloride (Figure 1D) as well as in the two enzymes alanine aminotransferase (Figure 2A) and aspartate aminotransferase (Figure 2B) in the serum of both sexes. The lowest values of sodium stem from various studies from two companies, while the highest values (≥169 mmol/L) are from a single study and exclusion could be justified. As there are other studies from two companies with ≥160 mmol/L, which also deviate from the usual range in the scatterplot, and there is no clear gap, exclusion of measurements would have been arbitrary and therefore the values were kept in the analysis. Notably, the study with the highest sodium concentrations contributes most of the lowest calcium concentrations (<2.1 mmol/L), where other studies from two companies are also involved. The highest calcium concentrations (≥3.5 mmol/L) come from four studies supplied by two companies and one of these studies contributes most measurements.

High alanine aminotransferase (Figure 2A) levels (≥3.0 μkat/L) originate from four companies and aspartate aminotransferase (Figure 2B) levels (≥5.0 μkat/L) from all five companies, indicating that these measurements are not based on erroneous entries. However, it should be noted that certain studies are more prevalent among these high values.

Although the scatterplot of triglycerides (Figure 1I) shows minimal differences between sexes, the means differ markedly (geometric mean 0.55 mmol/L for females and 1.02 mmol/L for males, see Supplementary Material).

**FIGURE 4:**
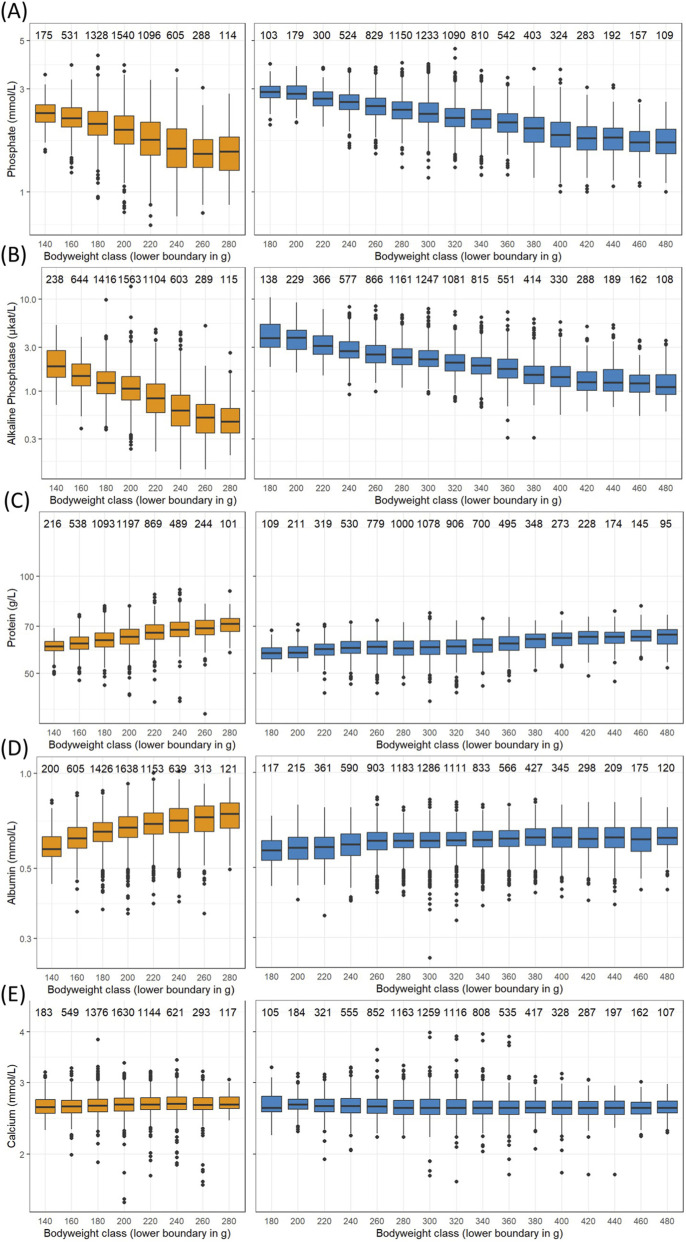
Box and whiskers plots of selected logarithmized measurements in serum versus female (left column) and male (right column) body weight classes from Han Wistar rats in the ViCoG DB; **(A)** Phosphate, **(B)** Alkaline phosphatase, **(C)** Protein, **(D)** Albumin, **(E)** Calcium. Boxes show the median and first and third quartiles and whiskers the range with separate dots for measurements more than 1.5 inter-quartile range off. The numbers above show the measurement counts within each class.

Certain parameters exhibit a correlation with body weight, serving as a surrogate for age. Most notable decreases are seen in serum phosphate (Figure 1E) and alkaline phosphatase (Figure 2C). Body weights were categorized into 20 g classes to show the changes (Figure 4). Phosphate levels decline with increasing body weights in both sexes (Figure 4A), as confirmed by mixed effect model ANOVA together with the company, study year and subject, where the likelihood ratio tests for the body weight classes were statistically highly significant (females: chi^2^ = 1562.2, df = 7, p < 0.001, males: chi^2^ = 4366.3, df = 15, p < 0.001). The same is true for alkaline phosphatase activity in serum (Figure 4B, females: chi^2^ = 2944.6, df = 7, p < 0.001, males: chi^2^ = 5060.8, df = 15, p < 0.001).

Conversely, serum protein levels in both sexes increase with body weight (Figure 4C), supported by the mixed effect model ANOVA (females: chi^2^ = 815.5, df = 7, p < 0.001, males: chi^2^ = 1014.8, df = 15, p < 0.001). Albumin levels also rise in females (Figure 4D, chi^2^ = 436.5, df = 7, p < 0.001) and less pronounced but still highly significantly in males (chi^2^ = 74.3, df = 15, p < 0.001). The only parameter without a statistically significant influence of the body weight classes is chloride in the females (chi^2^ = 11.4, df = 7, p = 0.12). Other parameters exhibit only slight variations across body weight classes that are nevertheless statistically significant, and that is true for calcium levels in serum of females and males (Figure 4E) as an example that would be otherwise regarded as rather constant (females: chi^2^ = 17.2, df = 7, p = 0.016, males: chi^2^ = 87.7, df = 15, p < 0.001). These differences may result from uneven data distribution over the whole range of body weights and slight differences that can be discerned in the occurrence of outliers (Figure 4). The other parameters in the mixed effect model ANOVAs, namely, the company that provided the data and the year when the study has been performed also had statistically highly significant influences on measurements.

The three factors body weight class, company, and study year were therefore analyzed regarding their effect sizes when controlling the influence of the other factors in the overall variability (Table 1). In both sexes, the study year emerged as the most important factor with a medium effect (mean effect size: 0.127 for females, 0.117 for males), followed by company (0.097 for females, 0.102 for males) and body weight class (small effect: 0.047 for females, medium effect: 0.089 in males). Notably, large effect sizes of body weight class imply medium or small effect sizes of company and year. As expected from the observations made before, alkaline phosphatase, phosphate, and protein in serum belong to the parameters with highest effect sizes for body weight class in both sexes. In addition, large effect sizes are observed in males for erythrocytes, reticulocytes, MCH, and MCV. There are medium and small effect sizes in other parameters (Table 1) and almost no effect of body weight class on certain cell counts, particularly monocytes and neutrophils in both sexes, as well as basophils in males and eosinophils in females.

## 4 Discussion

While it may not be feasible to identify all potential confounders in legacy studies and thereby narrow down the accuracy of the intervals, the data provide valuable insight into the stability of measurements during growth, with some parameters (e.g., calcium) remaining consistent across a wide range of body weights, while others (e.g., phosphate) show a strong response. Parameters with narrow acceptable ranges (e.g., sodium) displayed less variation in percentiles and tolerance intervals compared to those with broader ranges (e.g., bilirubin, Hall, 2017, see Supplementary Material).

Some median and geometric mean values for Han Wistar rats (see supplementary data) closely align with mean values published for Sprague Dawley rats (Petterino and Argentino-Storino, 2006) for parameters like calcium, sodium, and glucose, while marked discrepancies exist for others. Cholesterol values approach or exceed upper tolerance limits, and especially triglycerides differ markedly, with 0.3–0.46 μmol/L in both sexes of Sprague Dawley and geometric means in mmol/L in Han Wistar rats (see 0.55 mmol/L for females and 1.02 mmol/L for males above, factor >1000), indicating potential strain-specific differences. A decline of alkaline phosphatase has also been noted between 4- and 13-week studies of Sprague Dawley rats (Petterino and Argentino-Storino, 2006), while phosphate data for 13-week studies has not been provided in that publication and therefore cannot be compared.

Though both sexes were kept separate, the factors of body weight class, company, and study year had statistically highly significant influences on measurement outcomes. To discern their relative importance, partial eta squared was calculated and overall year of study performance (“study year”) was the most influential factor, followed by company providing the study data and body weight class. There are probably various contributions of individual influences that are then subsumed in the factors study year and company. It is important to note that these will need further elucidation if data originating from selected studies are to be used as virtual controls in other studies.

The factor company could be further categorized into sites or test facilities, which may overlap as certain CRO facilities are utilized by multiple companies. The study year may introduce various hidden confounders arising from the methods or procedures applied. Gurjanov et al. (2023) identified the choice of anesthesia, specifically CO_2_ versus isoflurane, as a hidden confounder affecting electrolyte values. A significant challenge is that this information is not consistently recorded in the raw data set, such as SEND, necessitating additional efforts for retrieval. This level of detail remains unaddressed in the ViCoG DB.

Depending on the clinical laboratory parameter analyzed, the body weight class has still a large effect and these results correspond to the effects visible in the scatterplots. In clinical chemistry measurements as exemplified in this study, body weight in both sexes has a large effect on phosphate and protein, followed by a medium effect of creatinine in males and albumin in females, and small effects of creatinine and albumin in the opposite sexes, as well as bilirubin, cholesterol, glucose, and triglycerides in both sexes and chloride and sodium in males only, whereas potassium and calcium showed almost no effects. The largest effect overall in both sexes is apparent in enzyme activity for alkaline phosphatase whereas aspartate aminotransferase has only a small effect and alanine aminotransferase marginally in the males. In hematology data, the males exhibit large effects in MCV, MCH, erythrocytes, and reticulocytes, where only small or medium (reticulocytes) effects can be discerned in the females, followed by other medium effects of lymphocytes and leukocytes in females, hemoglobin and platelets in males, and small effects in platelets, MCV, MCH, and hemoglobin and basophils in females, and lymphocytes, eosinophils, leukocytes, and MCHC in the males. Notably, there were no effects on neutrophils and monocytes in either sex, nor on MCHC and eosinophils in females, and basophils in males. As body weight classes serve as surrogates for age, these results provide clear instructions where age should be considered as an important factor and where it might be regarded negligible. Alkaline phosphatase, phosphate, protein, and creatinine have also been mentioned as age-dependent in the dog (Aulbach et al., 2019) and creatinine and phosphorus in male cynomolgus monkeys (Li et al., 2022) which emphasizes their more general variability during growth in mammals.

In summary, the retrospective analysis of laboratory parameters from legacy *in vivo* data underscores the necessity and added value of data sharing and curation for developing innovative tools such as virtual control groups. The laboratory parameters are used to start the establishment of virtual control groups. Future work will address other domains like incidence data from macro- and micropathological findings. The curated database can be used for different purposes. Other promising applications could include, for example, AI-based algorithms to detect subtle differences within the virtual control dataset. The database could also serve as a knowledge base to inform pathologists about rare findings and set them into context, e.g., with regard to susceptibility of species or strains. In the future, read-across approaches may also be established with the inclusion of treated animals in order to optimize the use of existing legacy data. Although the current data may not be annotated enough to establish clear criteria for virtual control group creation, it demonstrates that not all parameters contribute equally to variance and thus may not all require a rigorous monitoring. Further annotation and exploration are ongoing in the VICT3R project (Steger-Hartmann et al., 2025).

## 5 Conclusion

Our work contributes to an improved insight into the influence and relevance of study parameters on measurement variability, where body weight of animals at start of study is one of the most important parameters. In addition, we have also identified parameters that are less dependent on body weight. In these cases, the relative influence of other factors, namely, the company providing the study data, as well as the study year, is more important.

Our analysis will serve as critical guidance for selecting appropriate parameters which need to be controlled when utilizing historical control data to establish reference intervals or generate virtual control groups.

## Data Availability

The data analyzed in this study is subject to the following licenses/restrictions: dataset has been transferred to IHI VICT3R project. Requests to access these datasets should be directed to Thomas Steger-Hartmann, thomas.steger-hartmann@bayer.com.

## Abbreviations

ANOVA, analysis of variance; BW, body weight domain in SEND; BWORRES, Result or Findings as Collected; BWORRESU, Unit of the Original Result; BWTEST, Test Name; DM, demographics domain in SEND; eTOX, electronic toxicity; eTRANSAFE, Enhancing TRANslational SAFEty Assessment through Integrative Knowledge Management; IMI, Innovative Medicine Initiative; LB, laboratory measurements domain in SEND; LBCAT, Category for Lab Test; LBORRES, Result or Finding in Original Units; LBORRESU, Original Units; LBSPEC, Specimen Type; LBSTRESC, Character Result/Finding in Std Format; LBSTRESU, Standard Units; LBTEST, Test or Examination Name; LBTESTCD, Test or Examination Short Name; MCH, mean corpuscular hemoglobin; MCHC, mean corpuscular hemoglobin concentration; MCV, mean corpuscular volume; SEND, standard for exchange of nonclinical data; VCGs, virtual control groups; ViCoG DB, virtual control group database.
